# *oprC* Impairs Host Defense by Increasing the Quorum-Sensing-Mediated Virulence of *Pseudomonas aeruginosa*

**DOI:** 10.3389/fimmu.2020.01696

**Published:** 2020-08-04

**Authors:** Pan Gao, Kai Guo, Qinqin Pu, Zhihan Wang, Ping Lin, Shugang Qin, Nadeem Khan, Junguk Hur, Haihua Liang, Min Wu

**Affiliations:** ^1^State Key Laboratory of Biotherapy and Cancer Center, Collaborative Innovation Center for Biotherapy, West China Hospital, Sichuan University, Chengdu, China; ^2^Department of Biomedical Sciences, University of North Dakota School of Medicine and Health Sciences, Grand Forks, ND, United States; ^3^West China School of Basic Medical Sciences & Forensic Medicine, Sichuan University, Chengdu, China; ^4^Key Laboratory of Resources Biology and Biotechnology in Western China, Ministry of Education, College of Life Sciences, Northwest University, Xi'an, China

**Keywords:** *Pseudomonas aeruginosa*, *oprC*, virulence, pyroptosis, STAT3/NF-κB

## Abstract

*Pseudomonas aeruginosa*, found widely in the wild, causes infections in the lungs and several other organs in healthy people but more often in immunocompromised individuals. *P. aeruginosa* infection leads to inflammasome assembly, pyroptosis, and cytokine release in the host. OprC is one of the bacterial porins abundant in the outer membrane vesicles responsible for channel-forming and copper binding. Recent research has revealed that OprC transports copper, an essential trace element involved in various physiological processes, into bacteria during copper deficiency. Here, we found that *oprC* deletion severely impaired bacterial motility and quorum-sensing systems, as well as lowered levels of lipopolysaccharide and pyocyanin in *P. aeruginosa*. In addition, *oprC* deficiency impeded the stimulation of TLR2 and TLR4 and inflammasome activation, resulting in decreases in proinflammatory cytokines and improved disease phenotypes, such as attenuated bacterial loads, lowered lung barrier damage, and longer mouse survival. Moreover, *oprC* deficiency significantly alleviated pyroptosis in macrophages. Mechanistically, *oprC* gene may impact quorum-sensing systems in *P. aeruginosa* to alter pyroptosis and inflammatory responses in cells and mice through the STAT3/NF-κB signaling pathway. Our findings characterize OprC as a critical virulence regulator, providing the groundwork for further dissection of the pathogenic mechanism of OprC as a potential therapeutic target of *P. aeruginosa*.

## Introduction

The Gram-negative bacterium *Pseudomonas aeruginosa* is an important opportunistic pathogen that causes severe major cause of acute and chronic lung diseases in mammals. *P. aeruginosa* is the primary cause of acute and chronic lung infection, resulting in high mortality in patients with underlying conditions, such as cystic fibrosis (1). Upon *P. aeruginosa* infection, the pattern recognition receptors (PRRs) on the cell membrane of hosts recognize the corresponding pathogen-associated molecular patterns (PAMPs), such as lipopolysaccharide (LPS) and flagellin (2). Activated PRRs, including toll-like receptors (TLRs) and Nod-like receptors (NLRs), facilitate inflammasome assembly, caspase autocleavage, and mature IL-1β formation, as well as a type of rapid inflammatory cell death termed pyroptosis (3). Gasdermin D (GSDMD) is found as the pyroptosis executioner, which is activated by both caspase-1 and caspase-11/4/5 cleavage (4). Upon GSDMD activation, the pore in the plasma membrane causes cell lysis due to GSDMD oligomerization and ultimately IL-1β release, which is a highly inflammatory event (5).

*Pseudomonas aeruginosa* is notoriously resistant to antibiotics, which is facilitated by multiple factors including the highly impermeable outer membrane, the multiple drug efflux system (6, 7), mobile genetic elements (MGE) (8), etc. Furthermore, the list of multidrug-resistant (MDR) *P. aeruginosa* strains is rapidly growing, and new antibiotic development is urgently needed. Therefore, a thorough understanding of the pathogenic mechanisms of its virulence factors and their interactions with the host is required in order to invent new therapeutic strategies to control the infections by the MDR *P. aeruginosa* strains (9). These bacteria can survive under various growth conditions with vesicles from their outer membrane (OMV). A previous study (10) described the proteomic profiles of OMVs of *P. aeruginosa* biofilms and found that the outer membrane proteins OprC, OprD, OprE, OprF, OprH, and OprG were significant components of the OMV. OprC is one of the outer membrane porins responsible for channel-forming and copper binding (11). Then, researchers focused on the relationship between MDR and OprC in *P. aeruginosa* and revealed (12–14) that OprC was unrelated to meropenem, ceftazidime susceptibility, and imipenem diffusion.

Recent studies showed that the *oprC* expression level is involved in copper homeostasis (15). The essential trace element copper is the cofactor of oxidoreductases in *P. aeruginosa*. The copper enzymes, such as cytochrome c oxidase, lysyl oxidase, and ferroxidase, possess crucial physiological functions. Although copper is generally bound to proteins, an excess of free copper is harmful to the cell due to its redox properties (16). To maintain copper homeostasis, organisms generate a set of cytoplasmic copper-sensing regulators and transporters, including OprC. Research has shown that OprC-bound Azurin (a copper-containing redox protein released by *P. aeruginosa*) is essential for copper transport under copper-limited conditions (17).

Here, we analyzed how *oprC* deficiency affects *P. aeruginosa* pathogenicity compared to the wild type strain. We noticed that *oprC* deficiency reduced quorum sensing potential and impaired motility in the bacterium. Furthermore, infection by *oprC* deficiency strain diminished inflammasome activation, cytokine secretion, and transcription factor activity, as well as a significantly lower pyroptosis in host cells. Our findings revealed a novel crucial function of *oprC* in controlling pathogenic virulence activity, providing a basis to further advance the pathogenesis details of *oprC*.

## Materials and Methods

### Mice

C57BL/6J mice (6–8 weeks) were obtained from the Jackson Laboratory (Bar Harbor, ME, USA). All animal studies were approved by the Institutional Animal Care and Use Committee (IACUC) of the University of North Dakota and were performed in accordance with the animal care and institutional guidelines. The experimental procedures for animals, including treatment, care, and endpoint, strictly followed the Animal Research: Reporting *in vivo* Experiment guidelines (18).

### Cell Lines

Murine macrophage MH-S cells were obtained from the American Type Culture Collection (Manassas, VA, USA) and were cultured in Roswell Park Memorial Institute 1640 Medium (Thermo Fisher Scientific, Waltham, MA, USA) supplemented with 10% fetal bovine serum and antibiotics (penicillin and streptomycin) incubated in a 5% CO_2_ environment at 37°C (19).

### Inhibitor Treatment

STAT3 inhibitor V, stattic (sc-202818), and BAY (sc-202490) were obtained from Santa Cruz Biotechnology, USA. Stattic inhibits the activation of the STAT3 transcription factor by blocking phosphorylation and dimerization events. Stattic was resuspended in dimethyl sulfoxide (DMSO) to generate a 50 mM stock solution. A working solution (500 μM) was generated by diluting the stock solution in PBS (final concentration of DMSO: 1%). MH-S cells were treated with 10 μM of the specific STAT3 Inhibitor V, stattic, 30 min before infection. PBS/DMSO was added to each untreated well in order to perform vehicle controls (final concentration of DMSO, 1% in PBS). BAY inhibits the activation of NF-κB and the phosphorylation of Iκ-Bα. BAY was dissolved in DMSO to generate a 10 mM stock solution and diluted (1:1,000) in fresh medium before use. MH-S cells were treated with 10 μM BAY for 1 h before infection. DMSO was added to each untreated well as vehicle controls (20).

### Bacteria Preparation and Infection Experiments

The wild type *P. aeruginosa* strain PAO1, the Δ*oprC* mutant, and the complemented strain (Δ*oprC*/p-*oprC*) were described previously (17). Bacteria were grown for about 16 h in LB broth at 37°C with 220 rpm shaking. The bacteria were pelleted by centrifugation at 5,000 g. Cells were changed to antibiotic-free medium and infected by bacteria in a multiplicity of infection (MOI) of a 10:1 bacterium-cell ratio for 2 h. Mice were anesthetized with 45 mg/kg ketamine and intranasally instilled 2 × 10^7^ clonal-forming units (CFU) of PAO1 in 50 μL phosphate-buffered saline. Mice were monitored for symptoms and killed when they were moribund (18).

### ELISA and LDH Assay

Mouse TNF-α, IL-6, and IL-1β uncoated ELISA kits from Invitrogen (San Diego, CA) were used to measure cytokine concentration. Pierce LDH Cytotoxicity Assay Kit was used for the quantification of LDH released from the cell. Culture supernatants were collected at the indicated times after infection for ELISA and LDH analysis in accordance with the manufacturer's instructions (21).

### Immunoblotting

Mouse Abs against p-p65 (p-NFκB p65 Antibody [Ser 536]: sc-136548), ASC (ASC Antibody [B-3]: sc-514414), caspase-1(caspase-1 p10 Antibody [M-20]: sc-514), and β-Actin (β-Actin Antibody [C4]: sc-47778) were obtained from Santa Cruz Biotechnology (Dallas, TX). Rabbit Abs against p65 (NF-κB p65 [D14E12] XP® Rabbit mAb #8242), STAT3 (Stat3 [D3Z2G] Rabbit mAb #12640), and p-STAT3 (Phospho-Stat3 [Tyr705] [D3A7] XP® Rabbit mAb #9145) were obtained from Cell Signaling Technology (Danvers, MA). Gasdermin-D (Anti-GSDMD antibody [EPR19828] ab209845) was obtained from Abcam. NLRC4 (Cat# PA5-88997) was obtained from Invitrogen (Carlsbad, CA). NLRP3 Rabbit pAb (Cat# A12694) was obtained from ABclonal (Woburn, MA). The samples derived from cells and lung homogenates were lysed in RIPA buffer, separated by electrophoresis on SDS-PAGE gels, and transferred to nitrocellulose transfer membranes (GE Amersham Biosciences, Pittsburgh, PA). Proteins were detected by western blotting using primary Abs at a concentration of 1:200 (Santa Cruz Biotechnology) or 1:1,000 (Cell Signaling Technology, Abcam, Invitrogen, and ABclonal) and were incubated overnight. Labeling of the first Abs was detected using relevant secondary Abs conjugated to HRP (Rabbit anti-Mouse IgG [H+L] Secondary Antibody, HRP; Goat anti-Rabbit IgG [H+L] Secondary Antibody, HRP, Invitrogen), which were detected using ECL reagents (GE Health) (22).

### RNA Isolation and Quantitative Reverse Transcription-PCR

Total RNA was extracted using TRIzol (Invitrogen) according to the manufacturer's instructions. For the mRNA assay, a total of 2 μg of DNA-free RNA was subjected to first-strand cDNA synthesis using the High-Capacity cDNA Reverse Transcription Kit (Applied Biosystems). The qRT-PCR assay was performed using PowerUp™ SYBR™ Green Master Mix (Applied Biosystems) and gene-specific primers (synthesized by Integrated Eurofins Genomics) in a CFX Connect Real-Time PCR Detection System (Bio-Rad). The separate well 2^−ΔΔCt^ cycle threshold method was used to determine the relative quantitative expression of individual mRNAs. Mammalian mRNAs were expressed as the fold difference to β*-actin*. Bacterial mRNAs were expressed as the fold difference to *16S* (23, 24).

### Histological Analysis

Lung tissues of three independent mice were fixed in 10% formalin (Fisher Scientific), soaked in 30% sucrose, and then embedded in optimal cutting temperature (OCT) compound. Six-micrometer sections were cut, stained by standard H&E protocol, and examined for differences in morphology after infection. The lung injury score for each sample was determined by neutrophil accumulation in the alveolar and interstitial space, formation of hyaline membranes, presence of proteinaceous debris in the alveolar space, and thickening of the alveolar wall. Each of these parameters was scored on a scale of 0 (absent) to 3 (severe) and summed to generate the lung injury score (25, 26).

### Swimming and Swarming

LB containing 0.3% (wt/vol) Difco agar (BD) was used for the swimming test. BM2 (62 mM potassium phosphate buffer [pH 7], 2 mM MgSO_4_, 10 μM FeSO_4_, 0.4% [wt/vol] glucose, and 0.1% [wt/vol] casamino acids) containing 0.5% (wt/vol) Difco agar was used for the swarming test. One microliter overnight LB cultures were introduced into the center of the agar plate by puncturing into the agar but without touching the base of the plates. The plates were incubated at 37°C for 24 h with the right side up. The diameter of the motility trace was measured (27).

### Twitching

LB medium supplemented with 1% (wt/vol) agar was inoculated by a tip stabbed through the agar to the agar-plastic interface, with 1 μL of cultures grown in LB broth. After 60 h of incubation, twitching motility was determined by measuring the diameters of the twitching zones stained by a 0.1% crystal violet solution (28).

### Measurement of Pyocyanin Production

Bacteria cultures were grown at 37°C, 220 rpm. Supernatants were collected after centrifugation at 10,000 rpm for 10 min and then filter sterilized. 4.5 mL of chloroform was added to 7.5 mL of supernatant and vortexed. Samples were centrifuged for 10 min at 10,000 rpm. The resulting blue layer at the bottom was transferred to a new tube. 1.5 mL of 0.2 M HCl was added to each tube and vortexed. Samples were centrifuged for 2 min at 10,000 rpm, and 1 mL from the pink layer was transferred to cuvettes. Spectrophotometric measurements were done at 520 nm. 0.2 M HCl was used as a blank. Pyocyanin concentration (μL/mL) was calculated by multiplying the value at 520 nm with 17.072 and then multiplying it again by 1.5 (27).

### Immunofluorescence

Collected lungs were embedded in OCT and were immediately frozen. Six-micrometer sections were cut using Leica CM1520 Cryostat. OCT was removed from cryosections in PBS, and the samples were fixed using 4% paraformaldehyde in PBS (pH 7.4) for 10 min at room temperature. Permeabilization and blocking were done in 5% BSA in PBS containing 0.25% Triton X-100. The expression of Claudin-1, ZO-1, TLR4, NLRP3, NLRC4, ASC, caspase-1, p-STAT3, and p-NFκB p65 was determined by immunofluorescence. Abs Claudin-1 (Invitrogen, Cat# 71-7800), ZO-1 (Proteintech, Cat# 66452-1-lg), TLR4 [Santa Cruz Biotechnology, TLR4 Antibody (25): sc-293072], NLRP3 (ABclonal, Cat# A12694), NLRC4 (Invitrogen, Cat# PA5-88997), ASC (Santa Cruz Biotechnology, ASC Antibody [B-3]: sc-514414), caspase-1 (Santa Cruz Biotechnology, caspase-1 p10 Antibody [M-20]: sc-514), p-NFκB p65 (Santa Cruz Biotechnology, p-NFκB p65 Antibody [Ser 536]: sc-136548), and p-STAT3 (Cell Signaling Technology, Phospho-Stat3 [Tyr705] [D3A7] XP® Rabbit mAb #9145) were used as primary antibodies at a 1:100 dilution. Goat anti-Rabbit IgG (H+L) Highly Cross-Adsorbed Secondary Antibody, Alexa Fluor 488 (Cat# A-11034, Invitrogen), or Goat anti-Mouse IgG (H+L) Cross-Adsorbed Secondary Antibody, Alexa Fluor 594 (Cat# A-11005, Invitrogen) was used at a 1:1,000 dilution as secondary antibodies. Cell nuclei were stained with DAPI solution (1 μg/mL DAPI in PBS). Slides were visualized with an Olympus FV3000 confocal laser scanning microscope. Quantification analysis was performed by Fiji (19).

### LPS Quantification Assay

Bacteria cultures were grown at 37°C, 220 rpm, until an OD600 of 0.5 was reached. Supernatants were collected after centrifugation at 10,000 rpm for 10 min and then filter sterilized. Diluted supernatants (1:4) were used for LPS measurement by Pierce LAL Chromogenic Endotoxin Quantitation Kit (Cat#88282 Thermo Scientific) in accordance with the manufacturer's instructions.

### Protease Assay

Bacteria were grown at 37°C, 220 rpm overnight. Supernatants were collected after centrifugation at 4,000 rpm for 30 min. 0.1 mL azocasein solution (30 mg dissolved in 1 mL water), 3 mL phosphate buffer (50 mM, pH 7.5), and 0.1 mL supernatant were mixed and incubated at 37°C for 1 h. Adding 0.5 mL 20% trichloroacetic acid (TCA) to stop the reaction. Supernatants were collected by centrifugation at 12,000 g for 10 min. Two hundred microliters supernatants were added to the microtiter plate for absorbance measurement at 366 nm (29).

### Alginate Assay

After bacteria had been cultured in 37°C shaker overnight, bacterial cultures were mixed with equal volume of 0.85% saline and centrifugated at 4,000 rpm for 30 min to collect the supernatants. The supernatants were mixed with equal volume of 2% cetylpyridinium chloride. The precipitates were collected by centrifugation at 4,000 rpm for 30 min. The precipitates were dissolved in 1 M HCl solution, precipitated with isopropanol, and dissolved again in the 0.85% saline. Fifty microliters samples were mixed with 200 μl of borate-sulfuric acid reagent (10 mM H_3_BO_3_ in concentrated H_2_SO_4_) and 50 μl of carbazole reagent (0.1% in ethanol) before incubation at 100°C for 10 min. Two hundred microliters of supernatants were transferred to the microtiter plate and absorbance at 550 nm was determined spectrophotometrically (30).

### Rhamnolipid Assay

Bacteria were grown in 5 mL LB-MOPS medium (dissolve 25 g LB powder and 10 g MOPS in 1 L deionized water, adjust pH to 7.2 using NaOH) overnight at 37°C, 220 rpm. After centrifugation at 4,000 rpm for 30 min to collect supernatants, 1N HCl was added to 4 mL supernatants to adjust pH to 2.3. Mixing 4 mL supernatant with 4 mL ethyl acetate and vertexing vigorously. After centrifugation at 500 rpm for 1 min, the upper phases were transferred to new tubes and evaporated to dryness. Methylene blue solution (Cat#1808, Sigma-Aldrich) was diluted 1:25 in deionized water and was adjusted to 8.6 pH by adding 15 μl 50 mM borax buffer. Four milliliters chloroform and 400 μl diluted methylene blue solution were added to the tubes containing the dry extracts and vortexed vigorously. After incubation at room temperature for 15 min, 1 mL chloroform phase and 500 μl 0.2 N HCl were added to 2 mL microcentrifuge tube and vortexed 20 s. The tubes were centrifuged at 500 rpm for 1 min. Two hundred microliters upper phases were transferred to the microtiter plate for absorbance measurement at 638 nm against an 0.2 N HCl blank (31).

### Growth Curves

The bacteria cultures were diluted when an OD_600_ value of 0.05 was obtained. The growth curves were performed in polystyrene microtiter plates by adding 100 μL cultures and incubated at 37°C. The optical densities at OD_600_ were recorded every 1 h (32).

### Flow Cytometry

Single cells were obtained from lungs digested by collagenase. The cells were stained for 1 h with abs PE Rat Anti-Mouse F4/80 (BD Pharmingen Cat# 565410), PE/Cy7 Anti-Mouse/Human CD11b (BioLegend Cat# 101215), PerCP/Cyanine5.5 Anti-Mouse CD45 (BioLegend Cat# 103132), and FITC Anti-Mouse Ly-6G/Ly-6C (Gr-1) (BioLegend Cat# 108406) diluted in PBS at a 1: 1,000. For compensation, single stained samples were set. Cells were analyzed on BD FACSymphony (BD). Data were generated using FlowJo V10 (Treestar, Stanford, CA).

### Statistical Analysis

Survival differences and growth curves were analyzed by the Kolmogorov-Smirnov test. In all other cases, one-way ANOVA with a *post-hoc* Tukey test was performed. For all statistical analyses, the statistical package R 3.6.0 was used.

## Results

### *oprC* Deficiency Impacts Bacterial Motility

**Figure 1:**
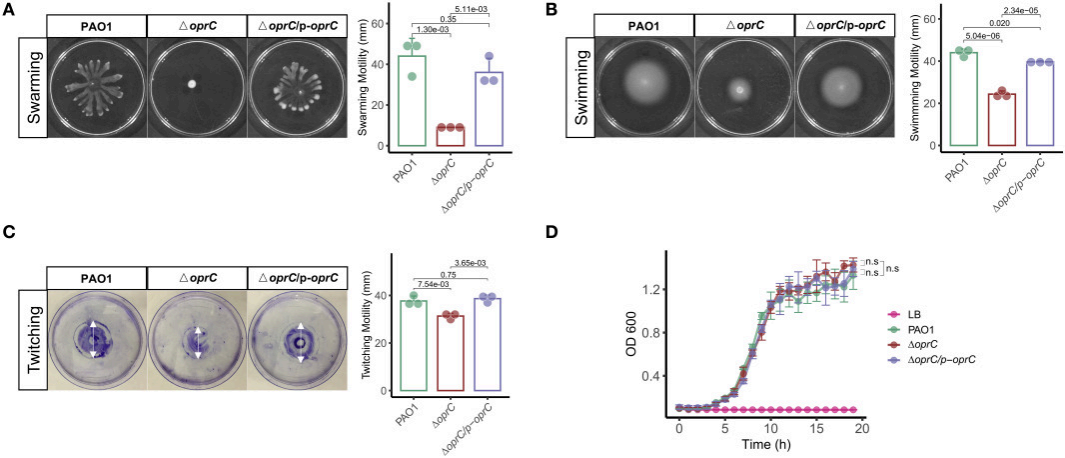
Altered bacterial motility in the Δ*oprC* strain. After the bacteria were incubated at 37°C for 24 h, **(A)** swarming motility (*n* = 3) and **(B)** swimming motility (*n* = 3) were assessed in a BM2 plate containing 0.5% agar and an LB plate containing 0.3% agar, respectively. **(C)** Twitching motility (*n* = 3) after incubating for 60 h at 37°C. **(D)** Bacterial growth curves (*n* = 4) on LB media are shown. Error bars represent the mean ± s.d. Kolmogorov-Smirnov test was performed for bacteria growth curves analysis. One-way ANOVA with a *post-hoc* Tukey test was performed for comparison of means of groups in other cases.

To investigate the effects of *oprC* deficiency on bacterial physiologic and/or pathogenic characteristics, we compared the swarming, swimming, and twitching motility between PAO1, Δ*oprC*, and Δ*oprC*/p-*oprC* strains (17). Supplemental Figure 1 shows decreased mRNA expression of Δ*oprC* compared to PAO1 (*p* = 2.10e-05) and Δ*oprC*/p-*oprC* (*p* = 6.90e-10) strains. Swarming of *P. aeruginosa* is a multicellular motility action relating to the quorum-sensing system (QS) (33–35). QS signals may modulate the expression and production of hundreds of virulence factors and regulate multiple downstream effects (36). As shown in Figure 1A, Δ*oprC* lost the dendritic branch features on BM2 swarming plates, and the diameter of the swarming zone was reduced by more than three quarters compared to PAO1 and complemented strains. We examined the swimming motility on swimming plates to assess the individual cell motility by rotating flagella (37). The swimming zone diameter of Δ*oprC* was half of that of the WT strain (Figure 1B). Next, we also examined the twitching motility related to type IV pili (37). Figure 1C illustrates decreased twitching motility of Δ*oprC* compared to PAO1 (*p* = 7.54e-03) and complemented strains (*p* = 3.65e-03). However, no apparent change in growth was induced by the *oprC*-deficient mutation (Figure 1D). Altogether, these findings suggest that *oprC*-deficient mutation impaired bacterial motility.

### *oprC* Is Involved in Virulence Regulation

**Figure 2:**
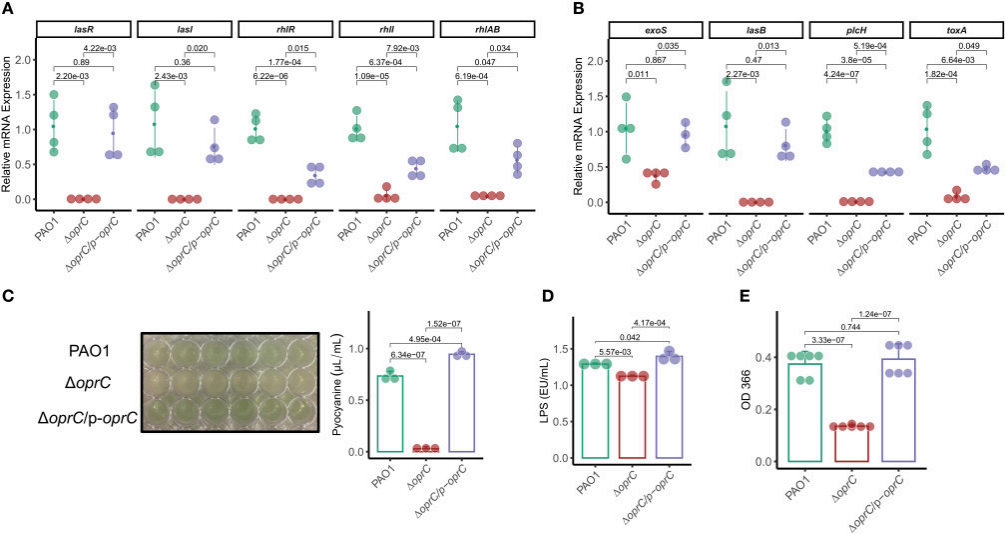
Δ*oprC* exhibited increased toxin production and upregulated virulence-related gene expression. **(A,B)** The expression levels of quorum-sensing and virulence genes were assessed by qRT-PCR (*n* = 3–4). **(C)** Quantification of pyocyanin production in *P. aeruginosa* (*n* = 3). **(D)** Quantification of LPS production in *P. aeruginosa* (*n* = 3). **(E)** Exoproteases activity of bacteria determined by the azocasein assay (*n* = 6). Error bars represent the mean ± s.d. One-way ANOVA with a *post-hoc* Tukey test was performed for comparison of means of groups.

The QS system is highly involved in competence, antibiotic production, biofilm formation, bacterial motility, and virulence factor secretion (36, 38). Given the bacterial motility changes of Δ*oprC*, we reasoned that QS system might be affected by the deletion mutation. We then measured expression levels of the genes known to be involved in the QS system. The *oprC*-deficient mutation significantly downregulated the expression of multiple QS system genes: *lasR* (*p* = 2.20e-03), *lasI* (*p* = 2.43e-03), *rhlR* (*p* = 6.22e-06), *rhll* (*p* = 1.09e-05), and *rhlAB* (*p* = 6.19e-04; Figure 2A). In addition, the expression of major virulence genes, such as *exoS, lasB, plcH*, and *toxA*, in the mutant strain was significantly decreased compared to PAO1 (Figure 2B). Next, we examined QS regulated virulence factors (38), pyocyanin (PCN), LPS, exoproteases, alginates, and rhamnolipids. PCN, a blue-green pigment mediating tissue damage and necrosis during lung infection, is one of the exotoxins secreted by *P. aeruginosa* (39). PCN secretion was drastically reduced in Δ*oprC* (*p* = 6.34e-07) compared to PAO1 and was reversed by *oprC* complementation (Figure 2C). LPS, also known as lipoglycans and endotoxins, were significantly reduced in the Δ*oprC* strain compared to PAO1 (*p* = 5.57e-03) and complemented strains (*p* = 4.17e-04; Figure 2D). The release of exoproteases, helping to dismantle the tissue connection (40), showed a similar pattern as shown in Figure 2E. Also, the productions of alginates and rhamnolipids (Supplemental Figures 2A,B) were decreased in the mutant group. Collectively, these results support that the *oprC* deletion mutant affects virulence regulation and toxin secretion of *P. aeruginosa*.

### *oprC* Deficiency Attenuates Mouse Mortality and Lung Damage Following *P. aeruginosa* Infection

**Figure 3:**
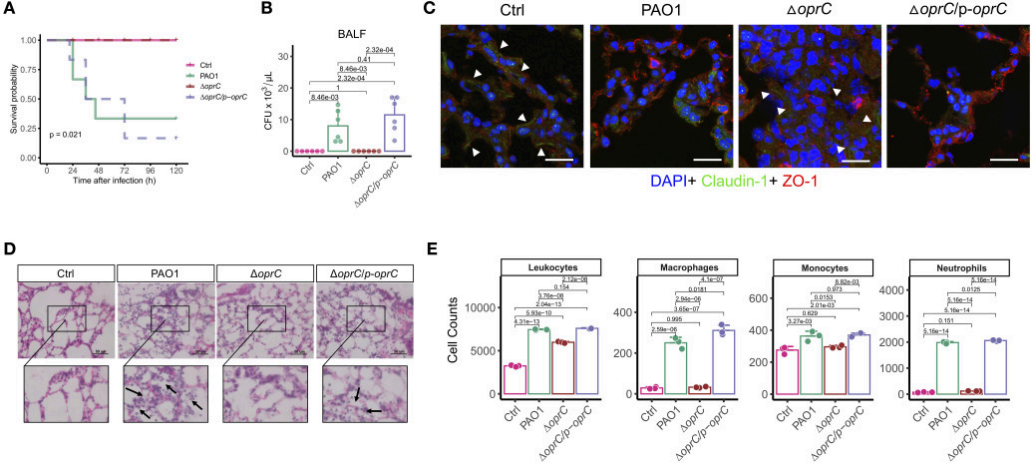
Δ*oprC* infection decreased mouse mortality and lung damage following *P. aeruginosa* infection. **(A)** C57BL6 mice were intranasally challenged with wild type PAO1, Δ*oprC*, and complemented strain at 2 × 10^7^ CFU in 25 μL PBS; moribund mice were killed to obtain survival data (*n* = 6). **(B)** Bacterial burdens in the BALF were determined 24 h after bacterial infection (*n* = 6). **(C)** Representative images of immunofluorescence staining of the lungs infected with bacteria for Claudin-1 co-stained with ZO-1 and DAPI (*n* = 3). Arrowheads show the membrane localization of Claudin-1. Scale bars, 20 μm. **(D)** Representative histological views of the lungs of mice 24 h after intranasal infection with bacteria by H&E staining (insets show the enlarged views) (*n* = 3). Arrows show examples of neutrophil infiltration areas. Scale bars, 50 μm. **(E)** Quantification of leukocytes, macrophages, monocytes, and neutrophils numbers per 30,000 cells collected from lung infected with bacteria for 24 h. Error bars represent the mean ± s.d. Kolmogorov-Smirnov test was performed for survival curves analysis. One-way ANOVA with a *post-hoc* Tukey test was performed for comparison of means of groups in other cases.

Due to the significant alterations in motility and virulence, we hypothesized that OprC potently affected the host-pathogen interaction. In an acute lung infection model, the *oprC*-deficient mutation completely protected the mice from death after infection compared to the PAO1 and the complemented strain (*p* = 0.021, Figure 3A). Mice infected with Δ*oprC* strain showed only lethargy within 12 h post-infection but recovered within 24 h post-infection, resulting in no death. Bacterial burdens were markedly decreased in the Δ*oprC* strain-challenged group compared to the PAO1 group at 24 h post-infection in bronchoalveolar lavage fluid (BALF; *p* = 8.46e-03), blood (*p* = 8.10e-03), and lungs (*p* = 2.14e-04; Figure 3B and Supplemental Figures 3A,B). In contrast to the Δ*oprC* strain-challenged group, there was no marked difference between PAO1 and complemented groups. As shown in Figure 3C, we noticed that change of Claudin-1 in localization from membrane to cytosol hampered the integrity of tight junctions in the PAO1 group (41), suggesting that PAO1 infection caused more severe lung barrier damages than ΔoprC strain. Also, the degree of lung inflammation in the Δ*oprC* strain-infected mice was significantly lower than that in the PAO1-infected mice (Figure 3D and Supplemental Figure 3C). Figure 3E showed more leukocytes, macrophages, monocytes, and neutrophils in PAO1- and Δ*oprC*/p-*oprC* strain-infected lungs. Gating strategies for flow cytometry were shown in Supplemental Figure 3D. Overall results demonstrate that *oprC* plays an important role in *P. aeruginosa* lethality in mice.

### *oprC* Deficiency Dampens Inflammatory Responses After *P. aeruginosa* Infection

**Figure 4:**
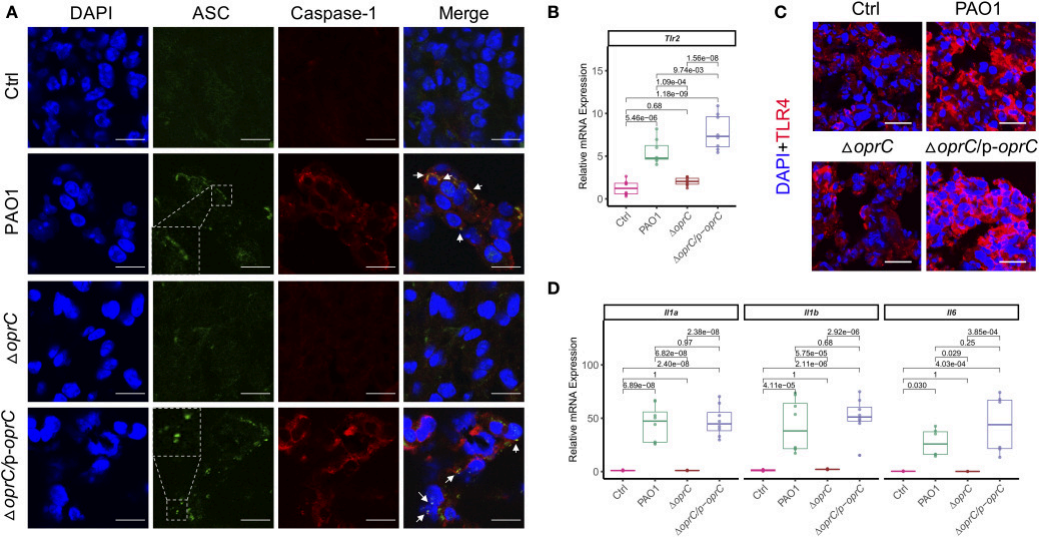
*oprC* deficiency dampened inflammasome formation and proinflammatory cytokines production upon *P. aeruginosa* infection. **(A)** Representative images of immunofluorescence staining of the lungs infected with bacteria for ASC co-stained with caspase-1 and DAPI (*n* = 3). Insets indicate the ASC speck-like structures. Arrows show the colocalization between ASC and caspase-1. Scale bars, 10 μm. **(B)** The RNAs were isolated from the infected lungs using TRIzol and reverse-transcribed into cDNA. The expression of *Tlr2* was assessed by qRT-PCR (*n* = 8). **(C)** Representative images of immunofluorescence staining of the lungs infected with bacteria for TLR4 co-stained with DAPI (*n* = 3–5). Scale bars, 20 μm. **(D)** The RNAs were isolated from the infected lungs using TRIzol and reverse-transcribed into cDNA. The gene expression levels of *Il1a, Il1b*, and *Il6* were assessed by qRT-PCR (*n* = 8). Error bars represent the mean ± s.d. One-way ANOVA with a *post-hoc* Tukey test was performed for comparison of means of groups.

NLRP3 and NLRC4 of the NLR family are the most widely studied inflammasomes activated by pathogenic organisms, including *P. aeruginosa* (42, 43). Real-time reverse transcription PCR (qRT-PCR) showed attenuated *Nlrc4* (*p* = 5.58e-06) and *Nlrp3* (*p* = 1.17e-08) gene expression in the Δ*oprC* strain-challenged lungs compared to PAO1-challenged lungs, whereas there was no apparent difference between the complemented and PAO1 strain (Supplemental Figure 4A). Immunoblotting results demonstrated increased expression of NLRC4, NLRP3, the adaptor protein ASC, pro-caspase-1, and cleaved caspase-1 p10 in PAO1-challenged lungs rather than the Δ*oprC* strain-challenged lungs (Supplemental Figure 4B). Moreover, we examined ASC speck formation in the infected lungs. Figure 4A showed more ASC specks and colocalizations between ASC and caspase-1 observed in PAO1- and Δ*oprC*/p-*oprC*-challenged lung sections but not in the Δ*oprC* sections, indicating that the inflammasome formation was downregulated by *oprC* deficiency mutation during *P. aeruginosa* infection. TLRs often serve as canonical sensors for various microbial component detection and innate immunity elicitation. TLRs, along with their adaptor proteins, initiate signaling cascades, leading to the activation of nuclear factor-kappa B (NF-κB) controlling the expression of inflammatory cytokine genes. Hence, we assessed *Tlr2* and *Tlr4* mRNA expression in infected and control lung tissue homogenates and found that *Tlr2* expression was markedly suppressed in the Δ*oprC* strain-challenged lungs (*p* = 1.09e-04; Figure 4B), while the gene expression of *Tlr4* was not significantly affected (Supplemental Figure 4C). However, the protein expression level of TLR4 was influenced by *oprC* during infection (Figure 4C and Supplemental Figure 4D). As NF-κB signaling activated by TLRs could initiate the transcription of various inflammatory cytokines, we next examined the expression of various cytokines in infected lungs. The mRNA levels of proinflammatory cytokines, including *Il1a* (*p* = 6.82e-08), *Il1b* (*p* = 5.75e-05), *Il6* (*p* = 0.029), *Il23a* (*p* = 8.33e-07), and *Il12a* (*p* = 1.68e-05), were significantly downregulated in the Δ*oprC* strain-infected lung tissues compared to the PAO1-infected group (Figure 4D and Supplemental Figure 4E). These data suggest that the inflammatory responses in the lungs infected with the Δ*oprC* strain were attenuated compared to the PAO1 group.

### *oprC* Deficiency Decreases Pyroptosis and STAT3/NF-κB Phosphorylation Following *P. aeruginosa* Infection

**Figure 5:**
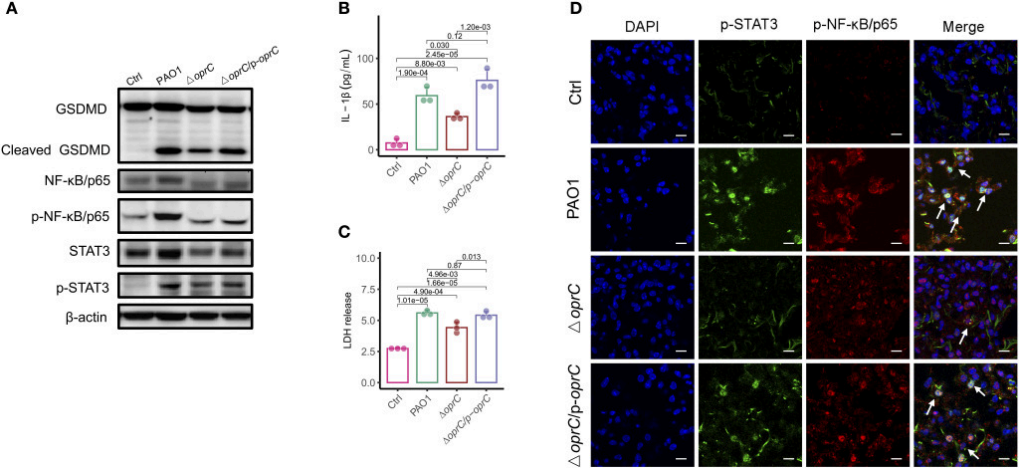
*oprC* deficiency decreased pyroptosis and STAT3/ NF-κB phosphorylation. **(A)** MH-S cells challenged with bacteria at a multiplicity of infection (MOI) of 10 for 2 h. Immunoblotting analysis of GSDMD, NF-κB/p65, p-NF-κB/p65, STAT3, and p-STAT3. **(B,C)** Secreted IL-1β and LDH from the supernatant was assessed by ELISA and LDH assay kit after MH-S cells were infected at an MOI of 10 for 2 h (*n* = 3). **(D)** Representative images of immunofluorescence staining of the lungs infected with bacteria for p-STAT3 co-stained with p-NF-κB/p65 and DAPI (*n* = 3). Arrows indicate the colocalizations between p-STAT3 and p-NF-κB/p65. Scale bars, 10 μm. Error bars represent the mean ± s.d. One-way ANOVA with a *post-hoc* Tukey test was performed for comparison of means of groups.

In response to inflammasome activation, GSDMD can be cleaved by caspase-1. The released N-terminal domain oligomerizes and creates plasma membrane pores that lead to pyroptosis and secretion of interleukin-1β (IL-1β) (4). We then examined whether the *oprC*-deficient mutation affects GSDMD cleavage and subsequent pyroptosis in MH-S cells (mouse alveolar macrophages). Figure 5A shows that infection by the mutant strain still induced GSDMD cleavage and pyroptosis but to a lower extent compared to PAO1 strain infection. Since the loss of membrane integrity results in the release of lactate dehydrogenase (LDH) and IL-1β into the extracellular space (5), we measured the release of IL-1β and LDH in MH-S cells. We found that both IL-1β and LDH in the Δ*oprC* strain-infected group were significantly reduced compared to the PAO1-infected group (*p* = 0.030 and *p* = 4.96e-03, respectively; Figures 5B,C). However, no significant change was observed between the complemented and PAO1 strain-infected groups in IL-1β and LDH release. Immunoblotting also showed decreased cleaved caspase-1 p10 and cleaved IL-1β in Δ*oprC*-infected cells compared to the PAO1 group, which is consistent with the results from the lungs (Supplemental Figure 5A). Furthermore, the STAT3/NF-κB signal pathway in the host has been shown to be activated to promote proinflammatory cytokine expression against *P. aeruginosa* infection (44, 45). The protein levels of STAT3 and NF-κB/p65 in MH-S cells infected with Δ*oprC* were markedly decreased (Figure 5A). Immunofluorescence staining of the infected lung sections showed that phosphorylation of STAT3 and NF-κB/p65 in the Δ*oprC* strain-infected lungs was not as strong as the PAO1 strain- or complemented strain-infected lungs (Figure 5D). We also found more colocalization between p-STAT3 and p-NFκB/p65 in the lungs infected with PAO1 or complemented strain (Supplemental Figure 4A). Overall, these results suggest that pyroptosis and STAT3/NF-κB activation during *P. aeruginosa* infection are impaired in Δ*oprC* strain infection.

### *oprC* Deficiency Attenuates Pyroptosis Dependent on Reduced STAT3/NF-κB Activation

**Figure 6:**
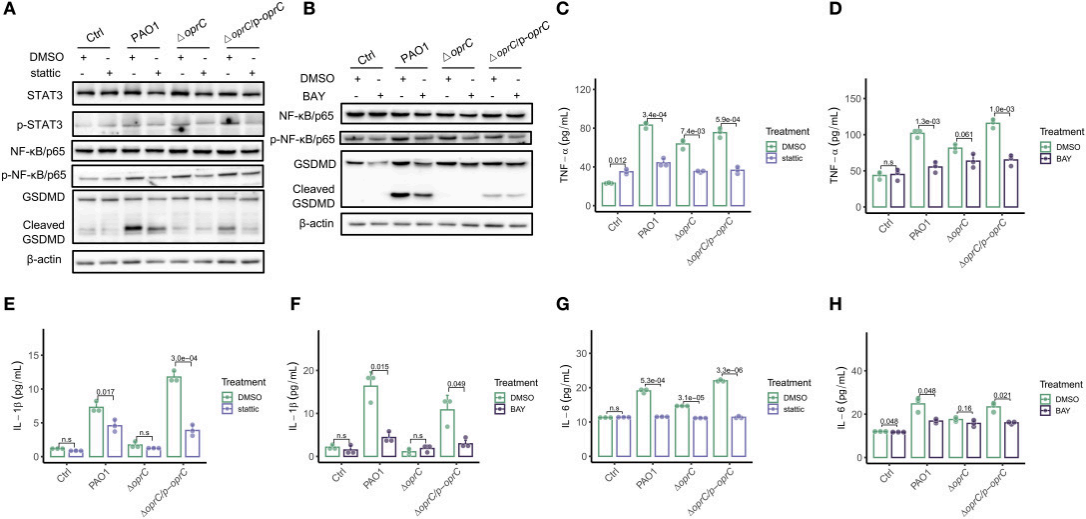
STAT3/NF-κB inhibitors decreased pyroptosis following *P. aeruginosa* infection. MH-S cells pretreated with 10 μm stattic (the inhibitor of Stat3 activation) for 30 min, or BAY (the inhibitor of NF kappa B activation) for 1 h, were challenged with bacteria at a multiplicity of infection (MOI) of 10 for 2 h. Cell lysis and supernatants were used for western blot and ELISA analysis, respectively. **(A,B)** Western blot analysis of STAT3, p-STAT3, NF-κB/p65, p-NF-κB/p65, and GSDMD. **(C–H)** TNF-α, IL-1β, and IL-6 were assessed by ELISA (*n* = 3). Error bars represent the mean ± s.d. One-way ANOVA with a *post-hoc* Tukey test was performed for comparison of means of groups.

To understand how *oprC* affects pyroptosis, we used chemical inhibitor stattic to block STAT3 phosphorylation and dimerization. We found reduced STAT3 phosphorylation, along with declined activation of NF-κB/p65 and GSDMD in the PAO1-infected and complemented-infected groups by the inhibitor, but not in the Δ*oprC* group (Figure 6A). Similarly, we used NF-κB inhibitor BAY to validate the data and noticed that BAY inhibited the phosphorylation of NF-κB/p65 and STAT3, as well as the GSDMD cleavage, only in the PAO1-infected and complemented-infected groups (Figure 6B). TNF-α is a major cytokine released by bacterial-pathogen-stimulated macrophages. STAT3 inhibitor administration significantly reduced TNF-α secretion in the Δ*oprC* group (*p* = 7.4e-03), as well as the PAO1 group (*p* = 3.4e-04) and the complemented group (*p* = 5.9e-04; Figure 6C). NF-κB inhibitor administration also reduced TNF-α secretion in the PAO1 group (*p* = 1.3e-03) and the complemented group (*p* = 1.0e-03) but only marginally in the Δ*oprC* group (*p* = 0.061; Figure 6D). In addition to TNF-α, phosphorylated NF-κB/p65 promoted expression of proinflammatory cytokines, such as IL-6 and IL-1β. We examined IL-1β secretion in the bacteria-infected MH-S cells, which showed that stattic and BAY pretreatment drastically decreased IL-1β production in MH-S cells infected with the PAO1 strain (*p* = 0.017 and *p* = 0.015, respectively) or the complemented strain (*p* = 3.0e-04 and *p* = 0.049, respectively) but not the Δ*oprC* strain (Figures 6E,F). The treatment with STAT3 and NF-κB inhibitors decreased the IL-6 secretion in the PAO1-infected group (*p* = 5.3e-04 and *p* = 0.048, respectively) and the complemented group (*p* = 3.3e-06 and *p* = 0.021, respectively) (Figures 6G,H). However, upon Δ*oprC* infection, stattic significantly reduced the IL-6 cytokine secretion back to the control level (*p* = 3.1e-05), while there was no significant difference in the BAY-treated group (*p* = 0.16). Collectively, these observations demonstrate that *oprC* deficiency attenuates pyroptosis, which is dependent on blunted STAT3/NF-κB activation.

## Discussion

Due to the growing antibiotic resistance, *P*. aeruginosa has increasingly become a major concern in hospital-acquired infections. These infections can occur in any part of the body with severe outcomes or death, imposing a heavy medical burden. The infections in the blood and lungs tend to be more severe and lead to pneumonia and/or bacteremia. The therapeutic strategies have been primarily developed based on controlling the critical virulence in order to kill pathogens, thereby reducing virulence, improving host immunity, and rescuing the infected patients.

We observed that the *oprC*-deficient mutation resulted in a change in bacterial motility. Despite no influence on bacteria growth, the *oprC* mutation diminished swarming, swimming, and twitching ability. Both multicellular swarming and individual swimming are bacterial motilities powered by rotating flagella, whereas twitching is mediated by the extension and retraction of type IV pili (37). Previous studies (46–49) showed that these three movements were positively associated with virulence factors, including the type 3 secretion system and its effectors, extracellular proteases, and iron transport.

Considering the important roles of the QS system in bacterial motility (38) and virulence modulation (50), we examined the transcription of two major QS systems, the LasR–LasI system and the RhlR–RhlI system (51). Interestingly, markedly declined expression of QS-associated genes (*lasR, lasI, rhlR*, and *rhlAB*) and typical virulence genes (*toxA, lasB, exoS*, and *plcH*) implies that *oprC* may participate in bacterial virulence regulation. Prior studies (52, 53) revealed that PCN is a crucial virulence factor of *P. aeruginosa* in the airway pathogenesis of cystic fibrosis patients. Furthermore, PCN has been shown to significantly enhance LPS-induced IL-1 and TNF-α release by monocytes (54). In this study, we noticed the marked reduction of PCN and LPS secretion in the mutant strain, as well as the further experiment results from exoproteases, alginate, and rhamnolipids, which indicated decreased virulence with *oprC* deficiency.

The changes in bacterial virulence should affect the host-pathogen interaction; however, how OprC impacts the host immune response is not well-known. Critically, our results demonstrated reduced mortality, lung barrier damage, and inflammatory responses in mice infected by the *oprC* deletion mutant. It was established that lung barrier integrity plays a critical role in homeostasis and immunity against pathogen invasion (55, 56). Once pathogen invades the host, the PRR will recognize the specific PAMP of the pathogen. The best-studied PRRs are the TLRs for the recognition of PAMPs of *P. aeruginosa*, including LPS, PGN, and flagellin (2). LPS recognition by TLR4 is universally attributed to triggering host defense responses against infection by Gram-negative bacteria, our data here indicated the decreased TLR4 expression in response to the *oprC*-deficient mutation of *P. aeruginosa*. Moreover, the gene or protein expression levels of inflammasomes (NLRP3 and NLRC4) and underpinning proinflammatory cytokines were assessed to probe the participation of inflammatory regulators. Consistent with previous studies (57, 58), both inflammasomes and inflammatory cytokines were activated during PAO1 infection. In contrast, *oprC* deficiency reduced inflammasome activation and the production of proinflammatory cytokines.

Generally, pyroptosis is a kind of cell death mediated by GSDMD. IL-1β and LDH can be released from the pore formed by active GSDMD. Meanwhile, IL-1β secretion is relevant to the inflammasome pathway, JAK/STAT, as well as the NF-κB signaling pathway. Our data showed the activation of GSDMD, along with the phosphorylation of STAT3 and NF-κB, caused by *P. aeruginosa*, but the activation was abolished by the *oprC* deficiency strain infection. We also observed the colocalization of p-STAT3 and p-NF-κB/p65 in PAO1-infected lungs, usually occurring in the cancer cells (59), which reflects potential interaction between STAT3 and NF-κB. NF-κB activated by TLRs can promote cytokine gene transcription, including Il-1β, and as feedback, Il-1β can in turn stimulate NF-κB activation (60). Similarly, IL-6 transcription can be regulated by the transcription factors NF-κB and STAT3. Moreover, IL-6 directly activates STAT3 (61).

The administration of transcription factor inhibitors (stattic and BAY) disrupted the positive loop and reduced the proinflammation cytokine secretion. The secretion of TNF-α, IL-1β, and IL-6 was sharply decreased after inhibitor treatment, while it was only slightly decreased in the Δ*oprC* group. Together with the alleviation of inflammation responses, the reduction of cleaved GSDMD results in the diminution of pyroptosis. Although no direct evidence was provided for STAT/ NF-κB facilitating GSDMD transcription, the upregulation of NLRP3 expression by NF-κB-dependent signals (20) supports the activation of GSDMD. Given the liaison between STAT3 and NF-κB (62, 63), blocking the function of either could decrease proinflammatory cytokine production and inhibit an excessive inflammatory storm in the host. *oprC* deficiency attenuates the inflammation response following *P. aeruginosa* infection via STAT3/NF-κB phosphorylation.

In summary, our study illustrates for the first time that OprC, which has recently been implicated in copper influx in *P. aeruginosa*, regulates the critical QS virulence signals and thereby strongly impacts the host immune response. It is not clear how OprC affects the QS, which may be related to copper as copper plays essential roles in cellular homeostasis maintenance as a co-factor for multiple enzymes. Here, our results demonstrate that *oprC* regulates the critical QS virulence signals, leading to a reduction in inflammasome activation, whereas exacerbated inflammatory responses profoundly impact cell viability, lung barrier integrity, tissue injury, and ultimately survival. Lung epithelial barrier is one of the critical mechanisms in preserving homeostasis and protecting immunity against pathogen invasion (55).

**Figure 7:**
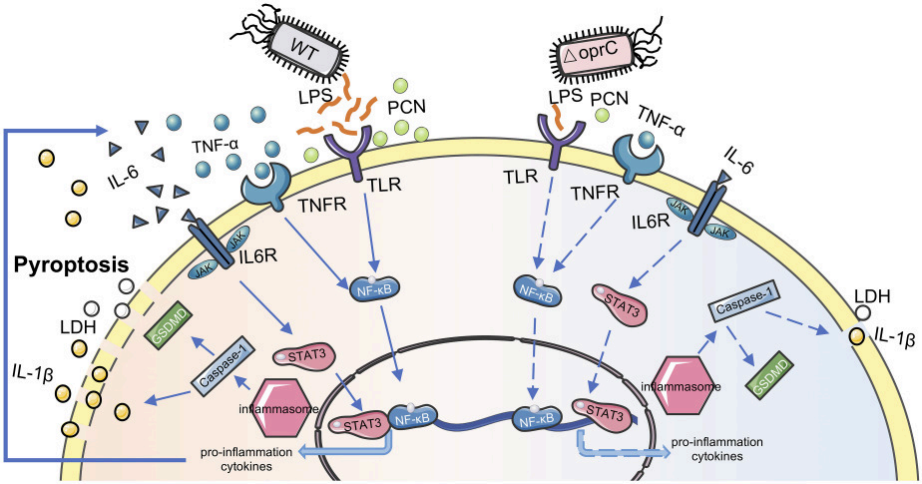
Proposed model for *oprC-*mediated virulence regulation. Compared with *P. aeruginosa* wild type PAO1, less virulent Δ*oprC* secretes less LPS and PCN, attenuating host receptor recognition following transcription factor activation. Less proinflammation cytokine secretion and weaker membrane recognition occur during Δ*oprC* infection. Finally, *oprC* deficiency decreases the pyroptosis induced by *P. aeruginosa*.

We proposed a model for the OprC-mediated virulence regulation and host immune response to *P. aeruginosa* infection (Figure 7). OprC triggers TLR signal activation by excessive LPS secretion, promoting NF-κB activation. Subsequently, with pore-forming protein GSDMD activated by caspase-1, pyroptosis is initiated, which represents rapid plasma-membrane rupture and release of proinflammatory intracellular contents. Cytokines released into the extracellular matrix elicit corresponding receptor recognition and transcription factor (STAT3 and NF-κB) activation. This positive feedback, often occurring after *P. aeruginosa* infection, is abolished under *oprC* deficiency conditions. *oprC* deficiency downregulates *P. aeruginosa* virulence, alleviates infection, and improves inflammation via reduced pyroptosis and STAT3/NF-κB phosphorylation. Importantly, our findings establish the critical virulence activity of *oprC* in physiological relevance in mice, shedding new light on the mechanistic understanding of *P. aeruginosa* pathogenesis and host-pathogen interaction.

## Data Availability Statement

All datasets presented in this study are included in the article/Supplementary Material.

## Ethics Statement

This animal study was reviewed and approved by Institutional Animal Care and Use Committee (IACUC) of the University of North Dakota.

## Author Contributions

PG, KG, QP, and MW designed the project and wrote the manuscript. PG, KG, QP, ZW, PL, SQ, NK, JH, HL, and MW revised the manuscript. PG performed most of the experiments with the assistance from ZW, QP, SQ, and PL. PG, KG, NK, JH, HL, and MW analyzed data. All authors contributed to the article and approved the submitted version.

## Conflict of Interest

The authors declare that the research was conducted in the absence of any commercial or financial relationships that could be construed as a potential conflict of interest.
