# Supplementary material for: oprC Impairs Host Defense by Increasing the Quorum-Sensing-Mediated Virulence of Pseudomonas aeruginosa
Source: Front Immunol. 2020 Aug 4;11:1696. doi: 10.3389/fimmu.2020.01696 (PMC7417366; doi:10.3389/fimmu.2020.01696)
Supplement: Supplementary file 1 [file Data_Sheet_1.pdf]

Supplementary Material

Supplemental Figures

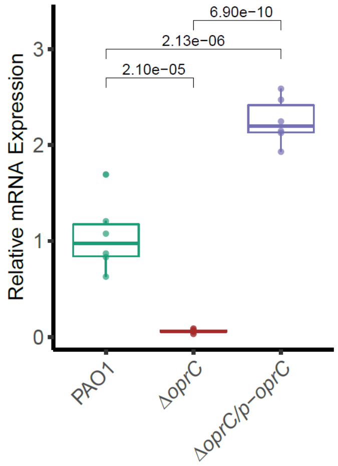

Supplemental Figure 1. Expression of *oprC* gene of bacteria assessed by qRT-PCR (n = 6). Error bars represent the mean ± s.d. One-way ANOVA with a post hoc Tukey test was performed for comparison of means of groups.

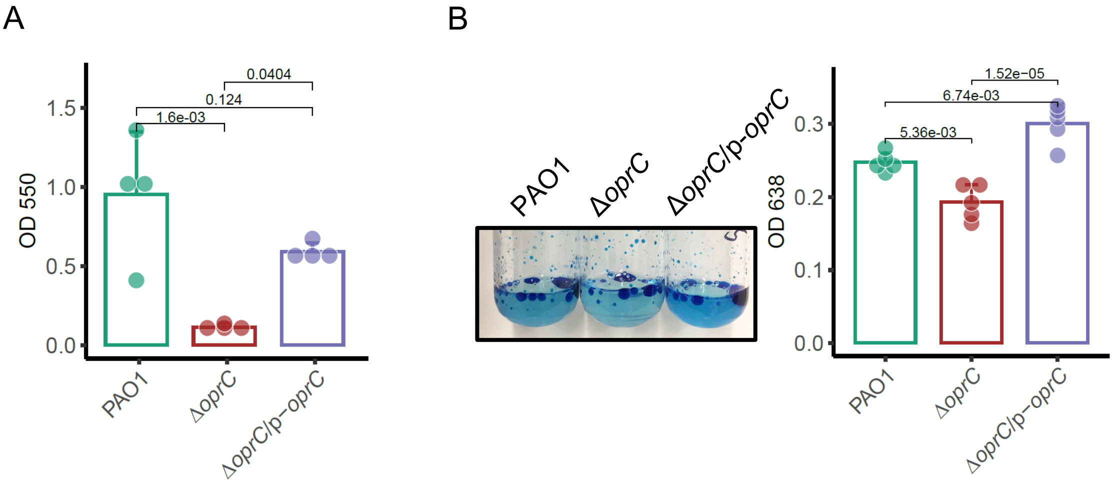

Supplemental Figure 2. (A) Alginates were extracted from bacteria and quantified by carbazole assay (n = 4). (B) Rhamnolipids measurement in the bacteria. Left, dry rhamnolipid-extract dissolved in chloroform-methylene blue complex. The blue color is proportional to the concentration of rhamnolipids. Right, rhamnolipids extraction solution was measured at 638 nm (n = 5). Error bars represent the mean ± s.d. One-way ANOVA with a post hoc Tukey test was performed for comparison of means of groups.

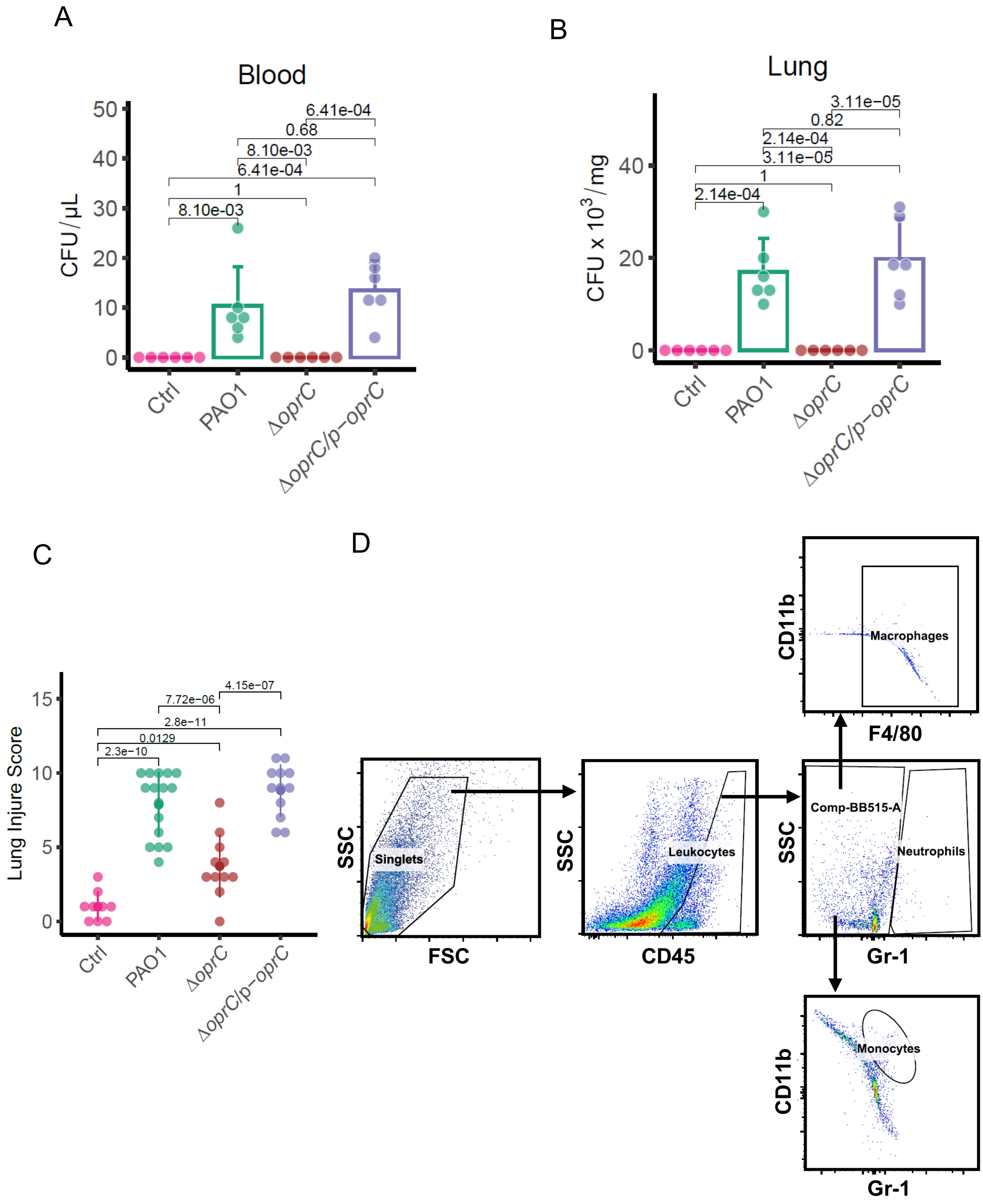

Supplemental Figure 3. (A-B) Bacterial burdens in the blood and the lungs were determined 24 h after bacterial infection (n = 6). (C) Lung injury score was evaluated and scored using a semiquantitative scoring system stated in the methods. (D) Gating strategies used in immune cell analysis from mouse lungs. Leukocytes ($CD45^{+}$), neutrophils ($Gr\text{-}1^{hi}$), macrophages($F4/80^{+}$), and monocytes ($Gr\text{-}1^{hi}$ $CD11b^{hi}$) were defined as shown. Error bars represent the mean ± s.d. One-way ANOVA with a post hoc Tukey test was performed for comparison of means of groups.

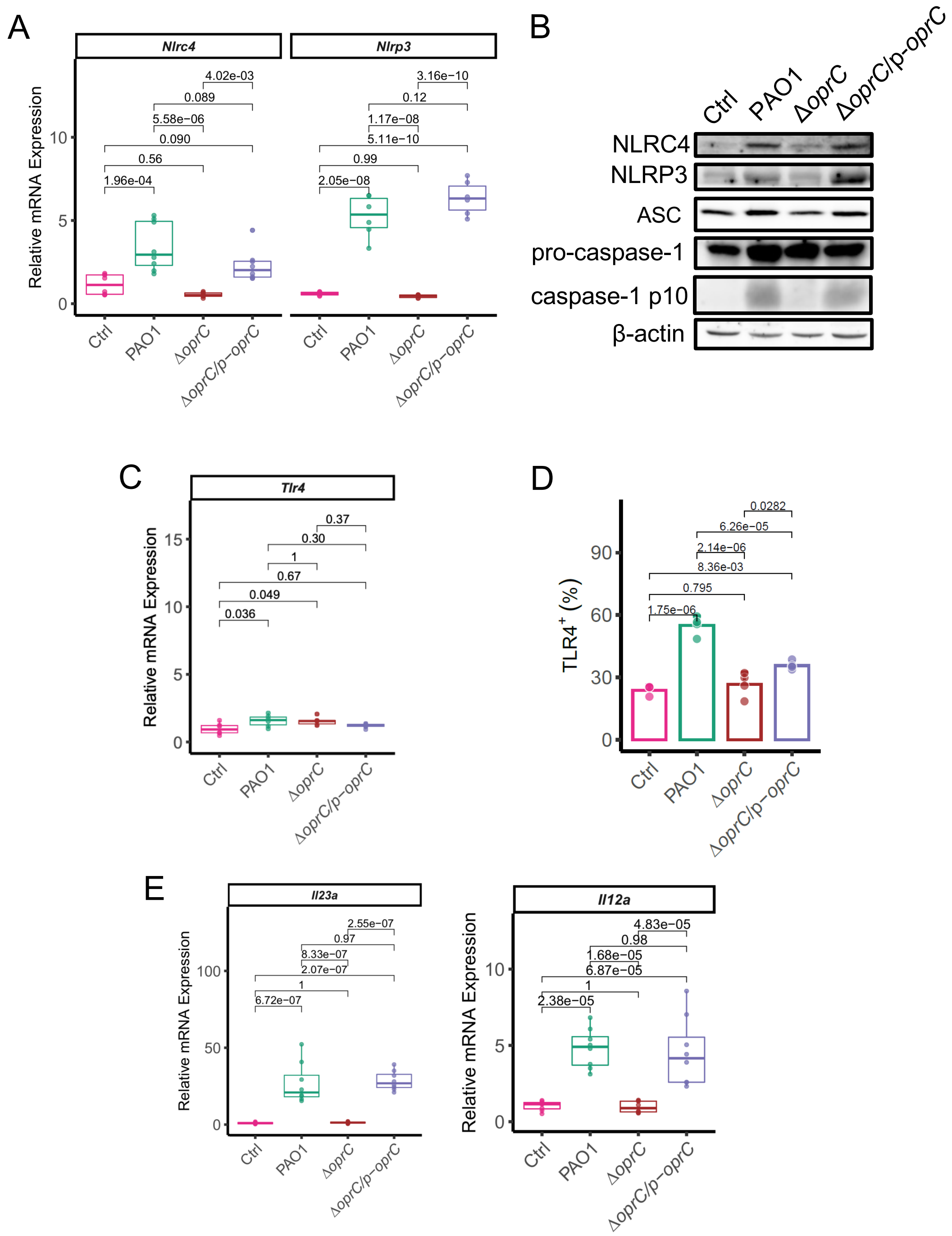

Supplemental Figure 4. (A) RNA was isolated from the infected lungs using TRIzol and reverse-transcribed into cDNA. Expression of *Nlrc4* and *Nlrp3* gene was assessed by qRT-PCR (n = 8). (B) Immunoblotting analysis of NLRC4, NLRP3, ASC, pro-caspase-1, caspase-1 p10 and β-actin from infected-lung tissue. (C) The RNAs were isolated from the infected lungs using TRIzol and reverse-transcribed into cDNA. The gene expression levels of *Tlr4* was assessed by qRT-PCR (n = 8). (D) Quantification of immunofluorescence staining of the lungs infected with bacteria for TLR4 co-stained with DAPI (n = 3-5). (E) RNA was isolated from the infected lungs using TRIzol and reverse-transcribed into cDNA. Expression of *Il23a* and *Il12a* genes was assessed by qRT-PCR (n = 8). Error bars represent the mean ± s.d. One-way ANOVA with a post hoc Tukey test was performed for comparison of means of groups.

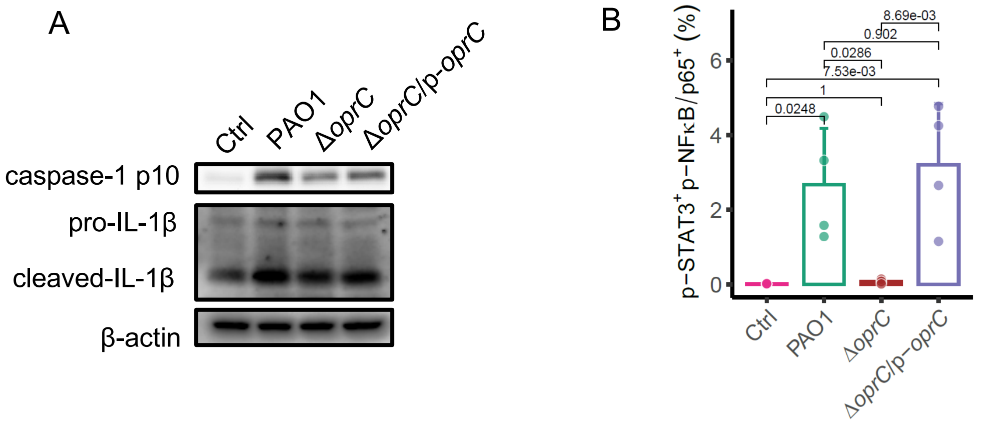

Supplemental Figure 5. (A) Immunoblotting analysis of cleaved caspase-1 p10, pro-IL-1β, cleaved-IL-1β, and β-actin from MH-S cell lines infected with bacteria. (B) Quantification of colocalization between p-STAT3 and p-NFκB/p65 in the lungs infected with bacteria (n = 4). Error bars represent the mean ± s.d. One-way ANOVA with a post hoc Tukey test was performed for comparison of means of groups.
