# Supplementary material for: oprC Impairs Host Defense by Increasing the Quorum-Sensing-Mediated Virulence of Pseudomonas aeruginosa
Source: Front Immunol. 2020 Aug 4;11:1696. doi: 10.3389/fimmu.2020.01696 (PMC7417366; doi:10.3389/fimmu.2020.01696)
Supplement: Supplementary file 2 [file Table_1.DOCX]

Supplementary Material

**Supplementary Table**

Supplementary Table 1. The primers used for qRT-PCR.

|  | **Forward (5’-3’)** | **Reverse (5’-3’)** |
| --- | --- | --- |
| *16S* | TGGTTTAATTCGAAGCAACG | ATCTCACGACACGAGCTGAC |
| *oprC* | GACGGCTCGCAGTTCAAG | GCTGACGTTCGATTTGACG |
| *lasR* | TGCCTAAGGACAGCCAGGACT | CCGAGGCTTCCTCGAAGAAC |
| *exoS* | ATCCTCAGGCGTACATCC | ACGACGGCTATCTCTCCAC |
| *lasI* | CGTGCTCAAGTGTTCAAGG | TACAGTCGGAAAAGCCCAG |
| *rhlI* | TTCATCCTCCTTTAGTCTTCCC | TTCCAGCGATTCAGAGAGC |
| *rhlR* | TGCATTTTATCGATCAGGGC | CACTTCCTTTTCCAGGACG |
| *toxA* | GGAGCGCAACTATCCCACT | TGGTAGCCGACGAACACATA |
| *rhlAB* | TCATGGAATTGTCACAACCGC | ATACGGCAAAATCATGGCAAC |
| *plcH* | GAAGCCATGGGCTACTTCAA | AGAGTGACGAGGAGCGGTAG |
| *lasB* | TTCTACCCGAAGGACTGATAC | AACACCCATGATCGCAAC |
| *Nlrp3* | ATTACCCGCCCGAGAAAGG | TCGCAGCAAAGATCCACACAG |
| *Nlrc4* | TTGAAGGCGAGTCTGGCAAAG | GGCGCTTCTCAGGTGGATG |
| *Tlr2* | GCAAACGCTGTTCTGCTCAG | AGGCGTCTCCCTCTATTGTATT |
| *Tlr4* | ATGGCATGGCTTACACCACC | GAGGCCAATTTTGTCTCCACA |
| *Il1a* | GCACCTTACACCTACCAGAGT | AAACTTCTGCCTGACGAGCTT |
| *Il1b* | GCAACTGTTCCTGAACTCAACT | ATCTTTTGGGGTCCGTCCAACT |
| *Il6* | TAGTCCTTCCTACCCCAATTTCC | TTGGTCCTTAGCCACTCCTTC |
| *Il12a* | CTGTGCCTTGGTAGCATCTATG | GCAGAGTCTCGCCATTATGATTC |
| *Il23a* | ATGCTGGATTGCAGAGCAGTA | ACGGGGCACATTATTTTTAGTCT |
| *β-actin* | GGTGTGATGGTGGGAATGG | GCCCTCGTCACCCACATAGGA |
